# Limitations in Contemporary Pharmacological Stroke Prevention Therapies in Atrial Fibrillation: A Descriptive Literature Review

**DOI:** 10.3390/jcm12206594

**Published:** 2023-10-18

**Authors:** Philippe Garot, Martin W. Bergmann

**Affiliations:** 1 Institut Cardiovasculaire Paris-Sud (ICPS), Hôpital Privé Jacques Cartier, Ramsay-Santé, 91300 Massy, France; 2 Asklepios Klinik, Altona, 22763 Hamburg, Germany; docbergmann@icloud.com

**Keywords:** atrial fibrillation, stroke, anticoagulants, prevention, atrium appendage, occluder device

## Abstract

The most common arrhythmia, atrial fibrillation (AF), increases with age and is associated with a 5-fold increased risk of stroke. Although lifelong oral anticoagulation (OAC) is strongly recommended for stroke prevention in patients with AF and CHA_2_DS_2_-VASc ≥ 2 only 50–60% of patients in Western countries belonging to this group are treated with oral anticoagulants, and less than half of these adhere to therapy over time. Before 2010, the numerous limitations associated with vitamin K antagonists (VKAs) were considered to be the reason behind OAC underuse; however, the approval of direct oral anticoagulants (DOACs) that require once- or twice-daily intake, no regular blood tests and fewer drug–food interactions has resulted in only modest improvements in OAC use and adherence.

## 1. Patient Non-Adherence to OAC Treatment

Very poor adherence with OAC treatment is a well-established phenomenon. The underuse and suboptimal adherence to OAC in AF is associated with increased risk of stroke. The concept of patient preference regarding OAC treatment should be viewed in the light of patient education and information about stroke and bleeding risks. This is key to improve long-term patient adherence to guidelines.

### 1.1. Incidence of Patient Non-Adherence

Approximately 50 to 60% of patients with AF and a CHA_2_DS_2_-Vasc ≥ 2 are treated with OAC, and less than half of these adhere to therapy over time [1]. There are different ways of not adhering to an OAC treatment. Treatment may be denied by the patient and never initiated, or treatment may be discontinued/interrupted with frequent cessation for ≥7 consecutive days and for various reasons, including medical or dental interventions, and finally prolonged or permanent interruption of treatment may also occur after, or not, an initial period of adherence. Interestingly, studies investigating patients’ non-adherence and OAC discontinuation have revealed varying rates of discontinuation across countries. Many of these studies were small, involved data from single countries, used different definitions of discontinuation, and were investigated over short time frames. Comparisons between countries are therefore complex. Reports from the Global Anticoagulant Registry in the Field-Atrial Fibrillation (GARFIELD-AF) showed the highest rates of discontinuation in the United States and South Africa [2]. Reports from the GLORIA-AF registry provide data for DOACs by region. Compared with Europe, discontinuation rates were higher in North America and Asia, while rates in Latin America and the Middle East were significantly lower. In GARFIELD-AF, insurance status and healthcare setting may have played a role [3]. Indeed, socioeconomic factors and local healthcare-related factors are likely to influence patient adherence.

### 1.2. Clinical Consequences of Patient Non-Adherence to Contemporary Pharmacological Stroke Prevention Therapies

The underuse and suboptimal adherence to OAC in AF is of concern, as continuous and consecutive use of OAC is crucial for stroke prevention; the risk of stroke increases by 7% per 10% decrease in the proportion of days covered by OAC, and gaps in OAC therapy of 1–3 months have been shown to double the risk of stroke in high-risk patients [4]. However, studies evaluating the association between OAC adherence and stroke risk have focused on OAC users, therefore excluding patients who have never initiated OAC therapy. Consequently, it remains unknown how the risk of stroke compares for patients who continuously adhere to OAC, versus non-adherent OAC users, versus non-users. Furthermore, it is unclear whether the stroke risk reduction associated with continuous adherence to OAC is similar for VKAs and DOACs. Although a recent report showed that adherence to OAC reduces the risk of stroke by nearly 40%; Medicare beneficiaries newly diagnosed with AF adhere to OAC on average for only one- third of the first year after the initial AF diagnosis [5].

The underuse and poor adherence with OAC remain a significant clinical challenge, whose mitigation would have a major impact on stroke prevention. In the GARFIELD-AF study, 13% of 23,882 patients discontinued OAC therapy for at least 7 days and had a higher risk of all-cause death (1.62 [1.25–2.09]), stroke/systemic embolism (2.21 [1.42–3.44]) and myocardial infarction (1.85 [1.09–3.13]) than patients who did not, regardless of whether OAC was resumed or not [2]. Importantly, the higher risk of an ischemic event after discontinuation was similar for patients treated with VKAs and DOACs.

### 1.3. Patient Preference

Based solely on the preferences of patients with AF, fewer patients would receive OAC treatment than would be expected based on recommendations. Consequently, AF patient preferences may be an important and potentially modifiable explanation for the often-observed undertreatment of patients with OAC. Therefore, it is of utmost importance not only to identify but also to understand patient preferences regarding OAC treatment in order to improve adherence. In addition, it is also important for the treating physician to educate and inform the patient about stroke and bleeding risks, as this is how adherence to guideline recommendations can be further improved. A difference in patient perception may exist regarding the treatment options, which may be particularly important as every treatment should be patient-centered. This is especially relevant in the long-term treatment of chronic diseases such as AF, as patient preferences may influence not only the long-term patient adherence, but also the physician–patient relationship and, ultimately, the actual effectiveness of a particular type of treatment. Consequently, it is important to know which OAC treatment characteristics patients prefer. This is in line with guidelines that strongly recommend taking patient’s opinions and preferences into account when deciding on OAC therapy options [6]. Stroke risk reduction and limited bleeding risk are the most important attributes for a patient when deciding whether they are for or against a certain treatment. In the stroke risk/bleeding risk trade-off assessment, physicians may be more sensitive to bleeding risk than patients. AF patients are willing to accept higher bleeding risks if a certain threshold in reduced stroke risk can be achieved.

## 2. Contraindications to OAC Therapy

The concept of contraindication to OAC treatment is well established, but there is no definition of what represents a contraindication to this long-term treatment in AF patients. In addition, there are several sources of bleeding for a given patient that may cumulate and improve the rates and severity of bleeding.

### 2.1. What Is a Contraindication to OACs?

Although the concept of contraindications to OAC therapy is well established, there is no clear or standard definition of what represents a definite or absolute contraindication to OAC therapies. It is noteworthy that contraindications to OAC therapies are usually reported as absolute if the bleeding risk significantly outweighs the potential benefit of the OAC therapies, and relative when the benefit/risk ratio seems more balanced. This is one of the reasons why this definition is not standardized; the benefit/risk ratio may vary depending on medical conditions, but also according to a patient’s profile, and its appraisal for a single patient may also vary from one physician to another. This is an individual definition, in which the severity of the bleeding lesion and the patient’s associated comorbidities are strongly intricated. Given that DOACs are associated with improved survival and decreased stroke rates in AF patients, some potential bleeding lesions that are candidates for resection should therefore be considered only as temporary contraindications to OAC therapies. In many other situations, the decision to interrupt or continue anticoagulants requires a global evaluation of the ischemic and bleeding risks and may be reconsidered after a temporary interruption and, for example, repeated brain imaging (DOACs resumption after intracranial bleeding). These strategies have been the focus of expert consensus, but despite some agreements on what is perceived as a contraindication to DOACs, many medical conditions and diseases that are considered absolute contraindications to DOACs for some specialists should be considered relative for others, and vice versa.

### 2.2. Sources of Contraindication to DOACs

**Figure 1:**
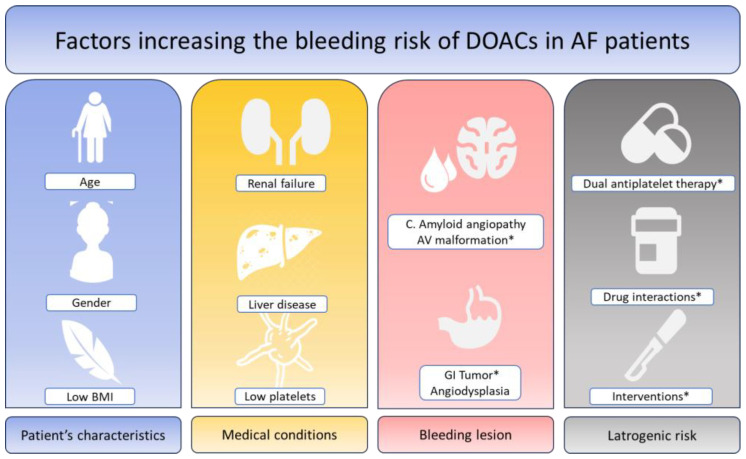
Factors increasing the bleeding risk of DOACs in patients with atrial fibrillation. DOACs: Direct oral anticoagulants. AF: Atrial fibrillation. * Modifiable factors or factors accessible to an efficient therapy.

Bleeding risk is a multifactorial condition in patients with AF. In addition, some of the conditions favoring bleeding may also increase the risk of ischemic events. There are schematically four different sources of bleeding with the potential to cumulate and further increase the rates and severity of bleeding: a lesion at risk of bleeding that may (or may not) be actionable; age and risk of falls; general conditions, including liver disease and renal failure; and iatrogenic risk, including antiplatelet treatment and drug interactions (Figure 1). Several lesions are associated with a high risk of bleeding including cancers and gastrointestinal benign tumors. Cerebral amyloid angiopathy (CAA) is a common finding in the aging brain, caused by the accumulation of amyloid-β peptide in the leptomeningeal arteries, cortical arterioles and capillaries, which is associated with a high risk of lobar intracerebral hemorrhage (accounting for 20% of all intracranial hemorrhages in adults). There is no specific therapy to treat these patients. Most neurologists consider CAA to be a contraindication to any anticoagulant or any antiplatelet agent. Gastrointestinal angiodysplasia is the second most common cause of lower GI bleeding in patients >60 years of age and is frequently associated with chronic bleeding. Endoscopic coagulation using heat probes, electric current or a laser is often insufficient in cases of recurrent bleeding episodes. Surgical resection is followed by bleeding in as many as 50% of patients. This is considered an absolute contraindication to DOACs by most GI practitioners. Many other lesions susceptible to bleeding are actionable and may require interventions and/or surgery to treat the disease and eliminate the risk of bleeding. Patient characteristics, including age, gender, low BMI, frailty, and risk of falls, are often associated with other conditions that increase the bleeding risk.

Among these, renal failure is associated with coagulopathy and severe liver disease with thrombocytopenia and a decrease in coagulation factors synthesis. The incidence of coronary artery disease increases with age, and AF patients are often predisposed to percutaneous coronary interventions that require dual antiplatelet treatment (DAPT) after the procedure. The so-called “triple therapy”, which consist of adding a DOAC to DAPT in AF patients is associated with HBR and is proposed to be shortened as much as possible in the guidelines, omitting aspirin immediately after hospital discharge [7]. Long-term treatment with DOACs is a major criterion in the Academic Research Consortium High Bleeding Risk initiative, which evaluated the risk of bleeding in patients requiring PCI [8]. Strategies to avoid bleeding in these patients are needed to mitigate the risk of DOACs without increasing the risk of stroke in patients at high-risk of recurrent myocardial infarction [9]. Analyzing prescription data from various European countries suggest that inadequate DOAC dosage reduction (particularly apixaban) when a bleeding risk is anticipated or observed is a growing practice by general practitioners; this comes at the price of an increased stroke risk as suggested by large-scale registry data [10].

## 3. Occurrence of Stroke under OAC

Although OAC therapies are given to AF patients in order to prevent the risk of stroke, the observed risk of embolic events is non-null. This phenomenon is probably more frequent when looking at the rate of non-overt or “subclinical” strokes. On the one hand, OAC acts on the embolic mechanism but does not have an effect on other risk factors of stroke; on the other hand, left atrial appendage closure may be a valid option with the potential limitation that another part of the atrium may be the source of thrombus formation.

### 3.1. Incidence

The use of DOACs for stroke prevention in patients with AF has generally improved patient care and treatment uptake. However, even in large, randomized control trials conducted in preparation for their approval, DOACs did not completely prevent stroke. In the ARISTOTLE study, the annual incidence of ischemic stroke was 1.0%, whether on VKAs or apixaban [11]. In addition, large, real-world registries observe an annual incidence of ischemic stroke of 1.56% in a group of 39,002 patients with a CHA2DS2-VASC of ≥4 [12]. Adherence to OAC therapy and higher stroke risk might influence this outcome. However, stroke units routinely treat patients with ischemic stroke despite DOAC therapy; CT or MRI imaging analyzing ischemic stroke patterns most often defines the cause of these strokes as “cardiac embolic”.

**Figure 2:**
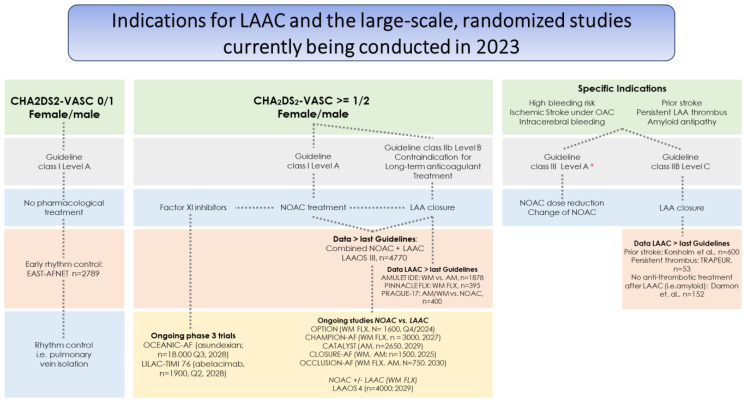
Stroke prevention in patients with AF. Indications for LAAC, and the large-scale, randomized studies currently being conducted in 2023. All studies were searched on clinicaltrials.gov, accessed on 8 August 2023 and the number of patients included as well as the data expected at the end of the study are displayed. AF: Atrial fibrillation [17,18]. * Modifiable factors or factors accessible to an efficient therapy.

This observation raises the question of whether DOAC therapy is sufficient to prevent thrombus formation in the LAA, which in the absence of mitral stenosis has been identified as the origin of cardiac thrombus in >95% of patients [13]. A meta-analysis of four randomized, controlled trials comparing DOACs to VKAs regarding LAA thrombus in transesophageal echocardiography in the context of cardioversion included 2397 AF patients. The study revealed a thrombus rate of 5% with no difference between DOACs or VKAs [14]. Predictors of LAA thrombus despite DOAC therapy include left atrial dilation, higher CHA_2_DS_2_-Vasc, severe mitral regurgitation and lower left ventricular ejection fraction [15]. To further improve stroke prevention, particularly in high-risk patients identified by CHA_2_DS_2_-Vasc, additional treatments regarding embolization of the LAA thrombus to the brain are needed. In fact, the recent LAAOS III trial showed that surgical closure of the LAA during CABG or valve surgery provided additional benefits to patients in terms of stroke incidence, independent of DOAC/VKA therapy, with a mean follow-up of 3.8 years and a mean CHA_2_DS_2_-Vasc of 4.2 [16]. The recently started large-scale, multicenter, international LAAOS IV trial is testing the hypothesis that percutaneous LAA closure with WATCHMAN FLX in addition to DOAC further reduces the occurrence of stroke—versus DOACs alone (Figure 2).

### 3.2. Subclinical Strokes: The Role of Brain Imaging

Over the past 20 years, clinically overt ischemic strokes have become a rare event during endovascular cardiac procedures, including LAAC, i.e., the prospective, multicenter, PINNACLE FLX trial of 400 patients using the latest generation WATCHMAN FLX device observed a 0.5% (n = 2) incidence of peri-procedural stroke with mild clinical sequelae. The prospective AMULET IDE trial recorded peri-procedural stroke with both the AMULET and the WATCHMAN Gen 2.5 device in 1799 patients; the event rate was <1% [19,20].

Subclinical ischemic lesions detected by sensitive brain and/or carotid imaging led to a higher incidence of events. In a prospective study of 54 patients during the M-TEER (mitral transcutaneous edge-to-edge therapy) procedure, continuous transcranial Doppler examination revealed micro-embolic signals in 9/54 (16.7%) patients, most often in cases of device–valve interaction [21]. Three Tesla brain MRI analyses within 24 h of LAAC in 23 patients detected acute brain lesions in 12/23 (52%), associated with a higher number of LAA angiographies. However, there was no change in NIHSS or MoCA neurological patient assessment when comparing pre-/post-procedure scores associated with these imaging findings [22]. Although it is plausible to improve outcomes, cerebral protection devices designed to capture debris during cardiac interventions have yet to confirm their clinical effect in transcatheter aortic valve implantation (TAVI) and other procedures such as LAAC [23,24].

### 3.3. Therapeutic Options

DOACs have been shown to reduce stroke incidence to a hazard ratio of 0.72 (95% CI 0.56–0.94) compared with no treatment, without increasing the risk of intracranial hemorrhage, not only in randomized studies but also in several real-world registries [25]. Extra-cranial bleeding under DOAC treatment is lowest with apixaban, while dabigatran and rivaroxaban lead to bleeding rates similar to phenprocoumon/warfarin in large registries [26]. Yet, no prospective head-to-head trial is available to prove the superiority of one DOAC over another in terms of efficacy or safety/bleeding events. Stroke risk under DOACs is associated with a higher CHA_2_DS_2_-Vasc score [27].

To further reduce the incidence of stroke, either more effective pharmaceutical approaches, potentially associated with higher bleeding risks, or a mechanical reduction in thrombus embolization from the heart is needed. The LAAOS III trial represents a milestone in the latter regard; 4670 patients with AF scheduled to undergo coronary artery bypass graft or valve surgery were randomized 1:1 to receive concomitant LAAC by various techniques. Most patients continued to receive OAC as indicated based on their CHA_2_DS_2_-Vasc score. At three years, 76.8% of patients continued to receive OAC. LAAC reduced the number of strokes or systemic embolism with a hazard ratio of 0.67 (95% CI from 0.53 to 0.85). In addition, 114 patients (4.8%) in the LAAC group and 168 (7.0%) in the “no-occlusion” group suffered a stroke or systemic embolism. LAAC at the time of cardiac surgery did not increase perioperative bleeding, heart failure or death [22]. This study confirms the concept that LAA is the main source of cardiac thrombi, as well as the benefits of LAAC in reducing these events; yet, stand-alone surgical LAAC has only limited data to support this approach [28]. The next step, the LAAOS IV trial, will enroll 4000 patients at 250 international sites on DOAC therapy in mid-2023. The study will randomize (1:1) patients between interventional LAAC using the WATCHMAN FLX in addition to continuous DOAC therapy vs. standard therapy alone. The safety and efficacy of this strategy will be tested for up to 4 years. The study will provide important data on whether LAAC, in addition to DOAC, reduces stroke and systemic embolism in patients with AF. Besides LAA, another part of the atrium is sometimes the source of thrombus formation in AF patients. This is one of the rationales of the LAAOS IV study, combining the benefits of anticoagulation on top of the mechanical occlusion of the LAA by the device.

Finally, some AF patients are still exposed to a severe course of ischemic stroke despite regular use of OAC because these drugs, apart from acting on the embolic mechanism, do not have a wider spectrum of effects on other risk factors [29].

## 4. Results in the Subgroups of Patients

DOACs have different efficacies in different subgroups of patients. The pivotal trials have excluded a non-negligible proportion of AF patients because of their clinical presentation or of clinical characteristics that could be perceived as a contraindication to the therapy.

### 4.1. Characteristics of Patients Recruited in the Trials

DOACs have been compared to VKAs in four large randomized clinical trials (RE-LY, dabigatran; ARISTOTLE, apixaban; ROCKET-AF, rivaroxaban; ENGAGE AF, edoxaban). To better understand in which population the results of these trials were achieved, we obviously need to look at the baseline characteristics of patients recruited in these trials, as well as the main reasons why they were excluded from participation; concerning the latter point, we lack data and evidence. The trials had many similar inclusion criteria, requiring the presence of AF to be documented by ECG. To be included in the RE-LY trial, patients had to have at least one of the following characteristics: previous stroke (but not recent), heart failure, age 75 or over or age 65 or over plus diabetes, hypertension, or coronary artery disease, all of which define a population at high risk of stroke. This situation was very similar in the other trials. The question of whether the protective effect of DOACs would have been as high as in the study in a younger population without prior stroke or heart failure remains unanswered. Reasons for exclusion were similar across the trials. Patients were ineligible if they had a recent stroke, severe valvular heart disease, and severe renal failure. Bleeding risk exclusions for recent trauma or major surgery, gastrointestinal bleeding, hemorrhagic disorders, and intracranial bleeding were well defined in the RE-LY, ROCKET AF, and ENGAGE AF trials. In the ARISTOTLE trial, patients with a bleeding risk perceived by the investigator as a contraindication to OAC were excluded. The reasons for exclusion in the trials were probably larger than current routine use, and the benefit-to-risk ratio remains uncertain in patients for whom we lack data and evidence. Strict adherence to the characteristics of patients included in the DOAC trial would probably limit the use of these drugs to a smaller proportion of the population.

### 4.2. Efficacy in the Subgroups

The prevalence of AF with valvular heart disease is increasing, and the coexistence of both diseases is associated with increased risk of thromboembolism. The landmark trials evaluating DOACs in AF patients only included a small number of patients with valvular AF, and those who had previously undergone bioprosthetic valve replacement or repair were excluded from the trials. A recent study examining the use of DOACs versus warfarin in patients with valve replacement/repair and AF showed that off-label use of DOACs in the United States is common, reaching 42% after mitral valve repair [30]. The study showed that DOACs are associated with a similar mortality in patients undergoing surgical and transcatheter bioprosthetic valve replacement, and reduced mortality in those undergoing surgical and transcatheter mitral valve repair. Compared to warfarin, ischemic strokes were more frequent with DOACs in patients undergoing bioprosthetic valve replacement and lower in those undergoing valve repair. Interestingly, major bleeding was less frequent with DOACs in both bioprosthetic valve replacement and valve repair cohorts. Future randomized controlled trials are warranted to determine the best anticoagulation strategy in patients with AF and concomitant valve disease.

There is little information on the safety and efficacy of DOACs in AF patients aged 90 years or older. The maximum age of included patients is not indicated in any of the available trails investigating DOACs, and it is unclear whether nonagenarians were included. There is an urgent need to collect information on the safety and efficacy of DOACs, which are increasingly prescribed to nonagenarians despite the lack of data. Given their known interaction profile and the possibility of monitoring these drugs, VKAs should be favored in nonagenarians until more data on the safety of DOACs are available. Although bleeding events were captured in these landmark trials, no subgroup analyses were performed to evaluate the characteristics of patients who bled. In the four landmark trials, most patients were elderly males, thus limiting the extrapolation of bleeding risk to other populations. In these studies, there was also no mention of bleeding risk scores. The lack of reporting on baseline bleeding risk, as well as the absence of subgroup analyses, creates a gap in understanding the potential factors that increase bleeding risk in patients on DOAC therapy. A recent analysis showed that most patients who experienced a bleeding event while on DOAC therapy were elderly, female, and overweight or obese [31]. Interestingly, these three characteristics encompass patients who have not been well-captured in trials.

A recent study indicated that anemia is common in patients with AF and is associated with major bleeding and a lower time range [32]. The authors reported that in these patients, OAC was associated with more major bleeding, and their protective effect on stroke and thromboembolism was significantly attenuated to the point where OAC no longer had any effect. The balance between the benefits and risks of OAC may be reversed in patients with moderate and severe anemia. There are several possible reasons why OAC therapies are not associated with reduced stroke. Patients with anemia have poorer anticoagulation control, and the benefits of OAC decline progressively in less time, possibly nonexistent once it is below 60%. In addition, a greater proportion of anemic patients have poor adherence with OAC compared to non-anemic patients. The possibility of more prominent hemorrhagic effects and increased vascular calcification in these patients might also play a role in this phenomenon. The question of whether patients with AF with moderate to severe anemia should not receive OAC remains unanswered, and physicians should carefully weigh the potential benefits and risks of OAC in these patients [32].

## 5. Factor-XI Inhibitors, the Holy Grail of OAC Therapy?

Although current DOAC treatment is effective in reducing the risk of stroke, extra-cranial bleeding is still an issue for a substantial proportion of patients. Dose reduction minimizes bleeding, yet also increases stroke risk. Inhibition of factor-XI rather than factor-X (apixaban, rivaroxaban, edoxaban) or factor-II (dabigatran) might reduce bleeding while effectively reducing the risk of stroke. Several Factor-XI inhibitors, either antibodies or antisense oligonucleotides, have been developed and are currently in different phases of clinical studies. Factor-XI inhibitors might reduce bleeding risk, as in theory they should only inhibit the intrinsic contact activation pathway without impairing hemostasis. The safety of Asundexian 20 mg and 50 mg once-daily, a small molecule inhibitor of factor-XI, was studied in the PACIFIC-AF phase 2 trial. Asundexian was compared to apixaban twice-daily in patients with AF and a CHA_2_DS_2_-Vasc of 2/3 in male/female patients, respectively. Patients were at increased risk of bleeding. Out of the 755 patients assigned to the three treatment arms, 3 patients on 20 mg asundexian, 1 patient on 50 mg asundexian and 6 patients on apixaban had a bleeding event, confirming a lower but still non-negligible bleeding risk with asundexian [33]. The ongoing OCEANIC-AF phase 3 trial (1st patient in 02/2023) is currently collecting data on the efficacy and safety of asundexian with regard to stroke rates and bleeding events in AF patients; the aim is to recruit 18,000 patients (until 08/2025) and provide clinical outcomes over a 3-year time frame (end of 2028, Figure 2).

Other approaches to inhibit factor-XI include antisense oligonucleotides (weekly subcutaneous injection) and monoclonal antibodies (monthly subcutaneous or intravenous injection). Although several approaches have been tested in phase 2 trials with the indication of deep venous thrombosis, only a small proportion of these have progressed to phase 3 trials (i.e., abelacimab) [34,35]. It is not yet known whether these drugs could replace the DOACs that will become generic over the next 2 years, or whether they will be restricted to high-bleeding-risk patients. It is unlikely that issues of patient adherence and the above-mentioned possible need for additional interventional measures to limit thrombus embolization from the LAA despite ongoing anticoagulant therapy will become irrelevant when factor-XI inhibitors are eventually approved in 4–5 years at the earliest.

## 6. Left Atrial Appendage Closure: Is a Limitation of OAC a Good Indication?

LAAC using interventional techniques started in the warfarin era. Early studies compared this approach to OAC with warfarin, showing equal efficacy and improved safety with regard to bleeding once procedure-associated risks, such as pericardial effusion, could be minimized through improved implantation techniques and devices. In 2012, this led to a Class IIA recommendation in the updated ESC guidelines for patients with an absolute or relative contraindication to OAC, even though this specific patient population had never been studied until then [36]. Several large-scale, prospective, multicenter registries have confirmed the safety and efficacy of WATCHMAN Gen 2.5 and AMULET in this group of patients [37,38]. As no new prospective, randomized, controlled multicenter trial were available in comparison to DOACs, LAAC was downgraded in the 2020 ESC guidelines to Class IIb, level of evidence B, restricted to patients with contraindication to long-term OAC [6]. In the US current guidelines, LAAC is recommended for all patients where long-term OAC is not a suitable option with a level of evidence IIA, B.

The first investigator-initiated trial comparing DOACs to LAAC was published in 2020. With only 402 patients randomized 1:1, the PRAGUE-17 trial lacked the power to test for stroke and bleeding events separately. The 4-year results published in 2022 confirmed the non-inferiority of LAAC with either WATCHMAN Gen 2.5 or AMULET to DOACs with regard to the combined endpoint of all-stroke/TIA/systemic embolism, clinically relevant bleeding, cardiovascular death, and procedure-/device-related complications in a cohort of AF patient with a mean CHA_2_DS_2_-Vasc of 4.7 ± 1.5 [39]. A nation-wide, propensity-matched Danish study showed the superiority of LAAC over DOACs in 600 AF patients with a history of ischemic stroke [18].

Currently, LAAC is the subject of a Class IIb level of evidence B recommendation (Figure 2) in patients who are not suitable for long-term OAC [6]. This is based on the results of prospective registries conducted in this patient population with the WATCHMAN and AMULET devices [37,38]. Recent randomized studies aimed to compare devices, namely the AMULET IDE trial for US approval and the PINNACLE-FLX trial for approval of the latest generation WATCHMAN FLX [20,40]. WATCHMAN FLX showed better results (vs. the prior generation WATCHMAN Gen 2.5) in terms of the incidence of peri-device leakage and device-related thrombosis with a similar safety profile, including both post-procedural DOAC treatment and dual antiplatelet treatment for up to 3 months [17,41]. Large-scale, multicenter, international, randomized studies comparing LAAC to DOAC in an all-comers AF population are currently recruiting patients, first results are expected for 2025 (Figure 2).

LAAC has also been studied in a number of specific patient cohorts not covered by any current guideline recommendations (Figure 2). Patients with excessive bleeding risk, i.e., with cerebral amyloid angiopathy, received a WATCHMAN or AMULET device without any post-procedural antithrombotic or anticoagulant therapy. Compared to patients receiving antithrombotic medications, this strategy was not associated with an increased number of device-related thrombi or strokes during or after the procedure. This confirms previous observations from the EWOLUTION registry, where the 1-year stroke risk was reduced when compared to the CHA2DS2-VASC-based expected rate [42,43]. LAAC therefore represents a viable option in these patients, as it does in patients with persistent thrombus (TRAPEUR registry) or patients with prior stroke on oral anticoagulation [18,42,44].

## 7. Conclusions

Despite recent improvements, contemporary pharmacological stroke prevention therapies employing DOACs still have significant limitations. Among these, bleeding events on OAC, other contraindications and patient non-adherence remain major concerns. In addition, DOACs have adverse results in certain subsets of patients and are not sufficiently effective to completely block LAA thrombus formation resulting in a non-negligible rate of strokes even under therapy. The new factor-XI inhibitors offer potential advantages regarding bleeding rates yet are not expected to increase effectiveness. European guidelines currently recommend left atrial appendage closure (LAAC) in patients with contraindications to OAC therapies. Major multicenter, controlled trials are underway to compare LAAC in an all-comers AF population to DOAC therapy as well as in addition to DOAC therapy. In part, these trials have completed patient recruitment and are already in follow-up (OPTION, CHAMPION-AF), with results expected for 2025. The data will possibly change the approach to stroke prevention in patients with AF.
